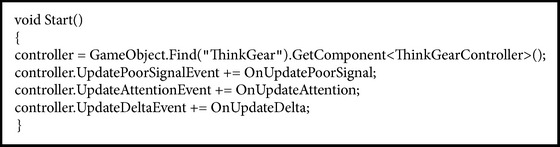# Novel Virtual Environment for Alternative Treatment of Children with Cerebral Palsy

**DOI:** 10.1155/2016/8984379

**Published:** 2016-06-14

**Authors:** Juliana M. de Oliveira, Rafael Carneiro G. Fernandes, Cristtiano S. Pinto, Plácido R. Pinheiro, Sidarta Ribeiro, Victor Hugo C. de Albuquerque

**Affiliations:** ^1^Programa de Pós-Graduação em Informática Aplicada, Universidade de Fortaleza, Avenida Washington Soares, 1321 Edson Queiroz, 60.811-905 Fortaleza, CE, Brazil; ^2^Instituto do Cérebro, Universidade Federal do Rio Grande do Norte, Avendia Nascimento de Castro 2155, 59056-450 Natal, RN, Brazil

## Abstract

Cerebral palsy is a severe condition usually caused by decreased brain oxygenation during pregnancy, at birth or soon after birth. Conventional treatments for cerebral palsy are often tiresome and expensive, leading patients to quit treatment. In this paper, we describe a virtual environment for patients to engage in a playful therapeutic game for neuropsychomotor rehabilitation, based on the experience of the occupational therapy program of the Nucleus for Integrated Medical Assistance (NAMI) at the University of Fortaleza, Brazil. Integration between patient and virtual environment occurs through the hand motion sensor “Leap Motion,” plus the electroencephalographic sensor “MindWave,” responsible for measuring attention levels during task execution. To evaluate the virtual environment, eight clinical experts on cerebral palsy were subjected to a questionnaire regarding the potential of the experimental virtual environment to promote cognitive and motor rehabilitation, as well as the potential of the treatment to enhance risks and/or negatively influence the patient's development. Based on the very positive appraisal of the experts, we propose that the experimental virtual environment is a promising alternative tool for the rehabilitation of children with cerebral palsy.

## 1. Introduction

Virtual environment is a technology able to establish a relationship between the user and the environment created, enabling real-time integration with controlled virtual objects. A virtual environment can be explored through visual and haptic devices, without real restrictions, for example, gravity. The iteration derives from the communication between human actions and the outcome of these actions, processed by the computer generating a response inside the virtual environment. The interaction can be passive, such as watching television, or active, when, for instance, users manipulate their body movements or a particular object inside a virtual scenario [1–3].

Virtual reality is a computational technology that provides artificial sensory feedback, allowing a user to experiment activities and events similar to those that could be found in real life and to develop motor abilities in three-dimensional (3D) virtual environments that resemble the real world [4]. Virtual reality involves three key elements that are required for motor learning: (i) repetitions, because neural plasticity is dependent on repeated stimulation able to produce optimal learning; (ii) sensory feedback, because intense multisensory stimulation is an essential part of rehabilitation for children with cerebral palsy, a systemic disease; and (iii) motivation of the patient [5].

Several studies have been carried out as an alternative tool for rehabilitation of patients with neurologic or genetic syndromes [6–15]. In most cases, the environment was based on games (serious game or exergames) applied to sensorimotor processing. The results are overall very promising, mainly due to the abstraction of traumatic symptoms such as pain and fear, to the escape from the real world, and to the incentive to overcome the challenges posed by virtual environments.

The use of games for clinical rehabilitation has boomed recently because of the availability of low-cost equipment on the market [16–25]. Another reason for the growing use of this type of treatment is the enhanced attractiveness of interactive environments in addition to the challenges posed by the game in pursuit of conquests/rewards (positive reinforcement) following the conclusion of a specific task. Virtual systems with clinical purposes have an important role in health care: they are easily manipulated by specialists as well as by patients, acting as a motivational source for continued treatment that is less aggressive and tedious than traditional treatments. It is worth emphasizing that the supervision of a clinical expert is extremely necessary for therapeutic success.

Games are incorporated into off-the-shelf commercial entertainment applications or specially developed for clinical purposes. Treatment of cerebral palsy in children is a challenging task for physicians. Lack of motivation and treatment withdrawal due to a delayed perception of patient's progress are two important factors that physicians have to deal with. The use of virtual environments may, thus, be an interesting approach as a complement to conventional treatment for these patients, establishing a new standard in the individual's rehabilitation strategy [6].

It is believed, according to the objectives of traditional treatment, that the rehabilitation process benefits from playful activities because of increased motivation and the reduction of environmental interference that may unfocus the child's attention to other actions which are not therapeutic targets. Given this background, the present work aims to develop a virtual reality game using Unity 3D version 5.2 (characterized as serious game) as an alternative tool of aid to motor and cognitive rehabilitation in children with cerebral palsy. We developed a virtual environment able to interact in real-time with children with cerebral palsy, via motion sensor of the hands and fingers (Leap Motion [26]), with the aim of using play to speed up recovery, by making patients feel more motivated, interested, and confident to carry out motor actions. Importantly, it is possible to perform actions in the virtual environment according to the specific needs of each patient without a direct intervention of specialist. Moreover, the combined recording of the electroencephalogram (EEG) through the device “MindWave” [27, 28] allows the tracking of the patient's clinical evolution in real-time, taking into account variations in the levels of attention and relaxation.

Evaluation of the performance of the proposed system was carried out by eight clinical specialists through a questionnaire that compared several aspects of the novel versus traditional treatments. Beyond this, specialists are submitted to the use of the proposed model, evaluating the potential of the tool, as well as suggesting possible changes or solutions.

A description of the virtual environment and its integration with Leap Motion and MindWave sensors is available at https://github.com/jullianamartins/ProjetoNami.

## 2. Experimental Procedures

Before the implementation of the new virtual environment, several meetings with clinical specialists (occupational therapists, physiotherapists, psychologists, neurologists, and pediatric neurologists) were performed at the Occupational Therapy Center of NAMI (Núcleo de Atenção Médica Integrada: Nucleus for Integrated Medical Attention) of the University of Fortaleza (UNIFOR), Fortaleza, Ceará, Brazil. During these meetings, we defined the age group of the patients from 0 to 8 years old, using as inclusion criterion the presence of major deficits in psychomotor development of the children. The different game phases were set to always increase in difficulty as the patient achieves a goal, that is, goes to the next phase. To model the virtual environment, we used an intuitive, efficient, and effective open source development platform called Unity.

### 2.1. Brief Description of Activities

After selecting the group of patients and during follow-up visits, we selected 6 activities, already present in the conventional treatment, to be modeled and used in the proposed virtual environment. These activities, divided into different levels to encourage the child to always overcome a goal (stimulating cognitive and motor domains), are called “functional play.” According to Wallon [29], these can be very simple movements such as flexing arms or legs, shaking fingers, touching objects, balancing the body, or producing sounds.

The purpose of each Phase is as follows:

1. *Phase 1*. Selecting all objects that contain the same color with the least amount of mistakes and in the shortest time possible. Distinguishing objects with different shapes and colors, developing motor abilities with hands and fingers, and using touch (pointing) through extension and flexing movements of the elbow joints.
2. *Phase 2*. Selecting all objects that contain the same shape, with minimal errors and in the shortest time possible. Distinguishing objects with different shapes, developing motor abilities through movement using small body muscles, and executing activities that require greater movement detail, such as writing, catching, and manipulating objects with their hands, moving the same joints as in Phase 1.
3. *Phase 3*. Selecting all objects that contain the same color and taking them to a basket of the same color, with minimal errors and in the shortest time possible. Distinguishing objects with different shapes but the same color, developing skills with hands through the extender and flexor movements of the elbow joint, and distinguishing laterality (right and left).
4. *Phase 4.* Identifying numbers and letters by dragging them to a basket according to their kind, with minimal errors and in the shortest time possible. Performing associations to distinguish letters and numbers, developing hand motor skills using the flexion and extension movements, and stimulating laterality by distinguishing right and left sides.
5. *Phase 5*. Selecting all objects that belong to the habitat being presented and taking them to a box. Distinguishing objects belonging to the same habitat according to spatial associations with rural or urban environments. Developing hand motor skills using the extensor and flexor movements of the elbow joint.
6. *Phase 6*. Supplementary and without the presence of explicit goals, with the aim of presenting the equipment used to create the virtual environment and of establishing a relationship between motor behavior and the objects in the proposed environment. Stimulating hand movements, through the extension and flexion of the elbow.

**Figure 1:**
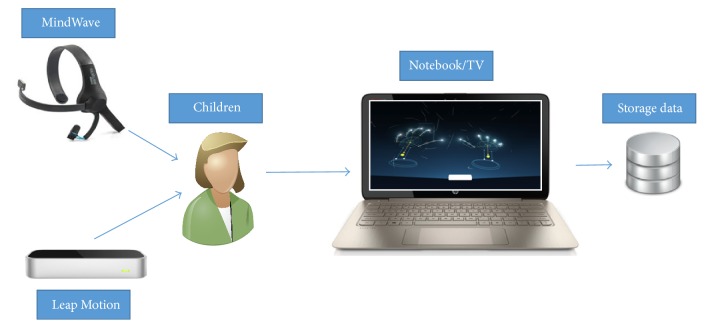
Interaction of patient/children/user with proposed virtual environment.

Please note that the integration of the child/user with the virtual environment occurs through the motion sensor of the hands and fingers (“Leap Motion”), in which it is possible to select and move objects to reach a specified goal, as explained above. Moreover, during the execution of the activities, the level of attention of the child/user is monitored by the EEG “MindWave” sensor, with the aim of correlating it with the patient's clinical evolution. Attention levels were measured through beta waves in the range of 13 to 30 Hz [30], captured every second during the Phase; when the Phase is completed, the sum of all levels of attention is divided by the execution time of the Phase, and, finally, the average of attention level is measured and stored. In this study, beta frequencies (equivalent to the level of attention [30]) were divided into low beta (13–16.75 Hz) and high beta (18–30 Hz), power was calculated in these bands, and then these values were associated with a range of 0–100 corresponding to different levels of attention of children. Value equal to 0 indicates that the ThinkGear is unable to calculate the level of attention, which can be due to excessive noise. Value between 1 and 20 denotes “strongly reduced” levels of the attention, indicating distraction, agitation, or mental abnormality. A value between 20 and 40 indicates “reduced” levels of the attention. Values from 60 to 80 are considered “slightly elevated” attention. Values from 80 to 100 are considered “elevated,” meaning they are strongly indicative of heightened levels of attention. The proposed system is presented in Figure 1.

### 2.2. Equipment Integration

For the development of this virtual environment, we used the framework Unity (5.5.2), in which the user is able to experience immersion in a computer-generated three-dimensional (3D) environment through specialized low-cost equipment.

Unity interacts with the user through the input devices: (i) Leap Motion sensor, in which hand movements are captured with millimeter precision, and (ii) MindWave sensor, which sends attentional feedback to the virtual environment by sending EEG signals in the form of a preprocessed string, so as to supply a range of attentional levels from 0% (no attention) to 100% (maximum attention).

The calculation to measure the level of attention from the EEG signals is based on the analysis of beta waves, as explained below. This information is extracted from a “socket connection” with a port and a standard local address, which allows data to be recorded in JSON format (communication protocol) and sent in real-time to the Unity. The same procedure was applied to the Leap Motion and its Software Development Kit (Leap Manage), which allows reading data in JSON and creating integration for various languages including a script (via plug or SDK) that can be interpreted by Unity. We achieved full integration of the data with the different pieces of equipment, allowing for interactions and enhanced controllability of the system.

#### 2.2.1. Leap Motion and Unity

Upload of the Leap Motion library to the Unity is performed through the command “using Leap.Util”; in Unity there is a set of classes aimed at the use of Leap Motion; it is necessary to import the package “*Assets*” with the command “*Unity CoreAsset*,” for this project was used at version 2.2.4 [31].

Adding a Controller object to the system, which serves as a connection with the Leap Motion service/daemon, is shown in Box 1.

The library's Leap Motion offers basic gestures, such as the following: (i) circle, which has the action of a finger braiding a circle, (ii) swipe, whose action has a long and linear movement of a hand and fingers, (iii) screen tap, which takes action of a move by tapping the finger to simulate touching a monitor vertically, and, finally, (iv) key tap, taking the action of moving by touching a finger, simulating touch of a keyboard key.  Box 2 represents the call for habilitation of these movements in the script.

#### 2.2.2. MindWave and Unity

The integration of MindWave with Unity is through the ThinkGear Connector (TGC), which uses host settings 127.0.0.1 (localhost), port 13845, and Transmission Control Protocol. Once the* socket* connection is established, the TGC captures the frequency data that the headset sends. When the SWF (Shockwave Flash) files open a socket connection, usually the SWF of this socket automatically prompts one TGC file, called crossdomain.xml, sending the XML in Box 3 to the TGC: "<policy-file-request/>" [32].

In response, the TGC will automatically write the XML in Box 3 for the socket to complete the approval of protocol.

In order for the information established through the protocol to be used by Unity, a new class is created for signal capture. The information captured for use in the application comprises the PoorSignal and Attention functions (see Box 4).

### 2.3. Animation

**Figure 2:**
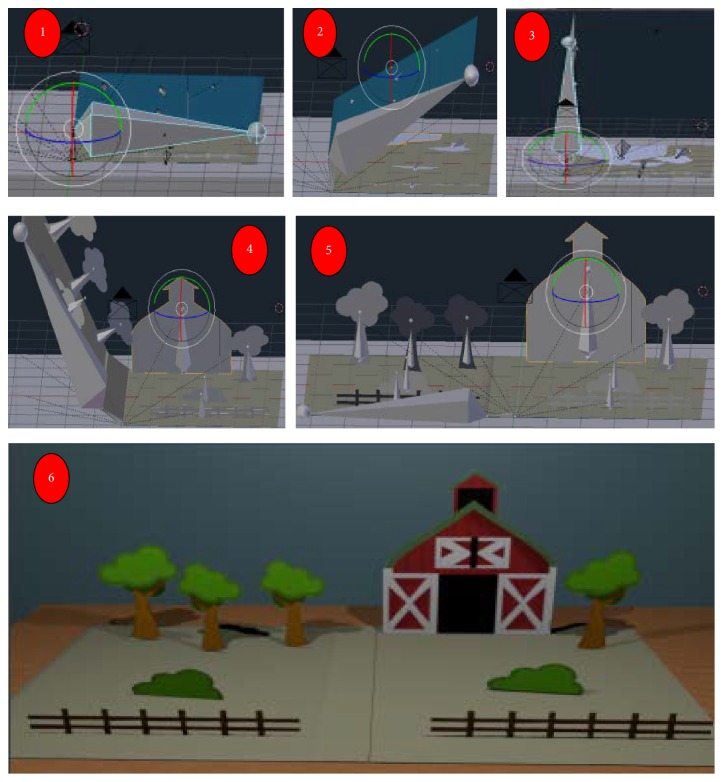
Construction and book opening animation and its rendered image.

The first contact of the child/patient/user with the proposed virtual environment is through animation of a book “opening” showing objects and the options to select which Phase the child will play as virtual treatment. The system saves the Phase executed during the last treatment session, as well as the end of each Phase, showing its completion. The animation is accomplished using a set of bones (skeletal representation used to animate objects) on the front of the book and the objects displayed, as shown in Figure 2, in which represent “opening” the book and its rendered image.

**Figure 3:**
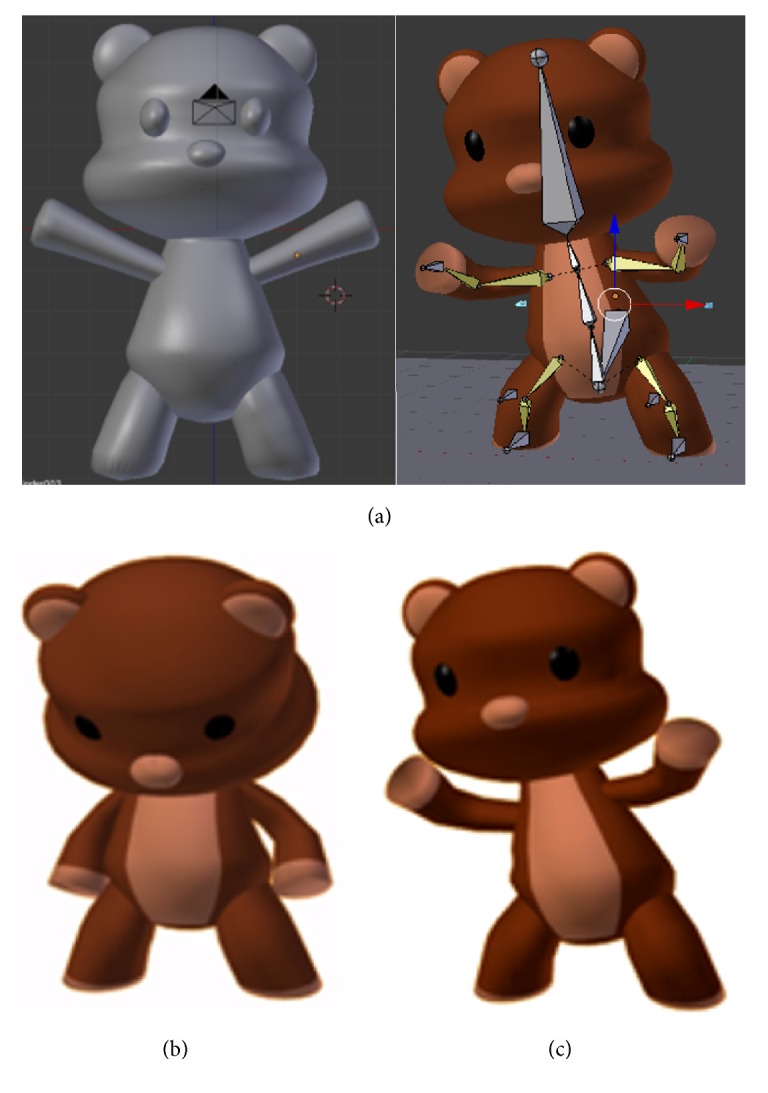
Bear (a) concepts and animation and its (b) sad and (c) happy representation.

After the animation of “closing,” corresponding to completion of a given Phase, a bear (Figure 3) with a happy or sad predefined expression is presented as positive or negative reinforcement, respectively, to the child. In order to prevent the child from becoming frustrated and from losing interest in the virtual environment, a specialist can assist them to successfully complete each Phase, increasing the interest for the next Phase.

## 3. Results and Discussions

**Figure 4:**
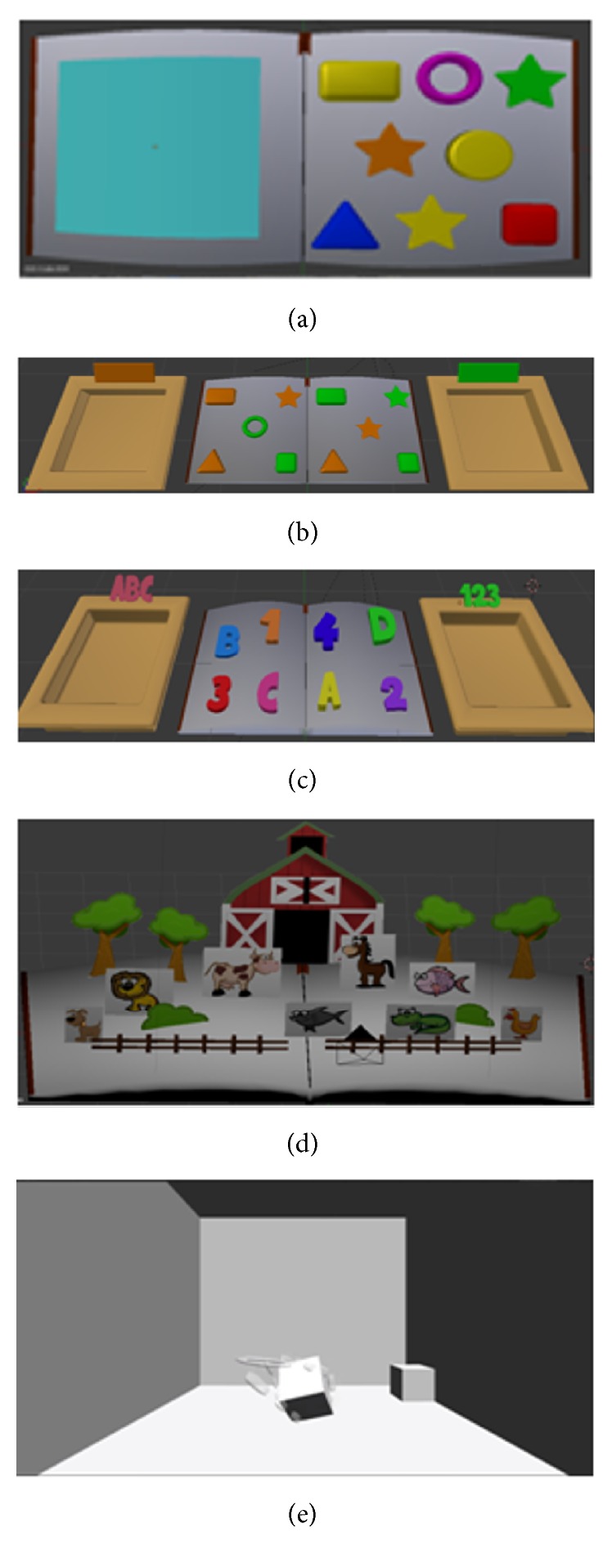
Screenshot of the virtual environment developed used in Phases (a) 1 and 2, (b) 3, (c) 4, (d) 5, and (e) 6.

*Modeling of the Activities*. The six scenarios corresponding to activities that each child will be submitted to, according to their clinical evolution or knowledge previously acquired through the conventional treatment, are presented in Figure 4. The goal of each Phase was described in Section 2.1.

**Figure 5:**
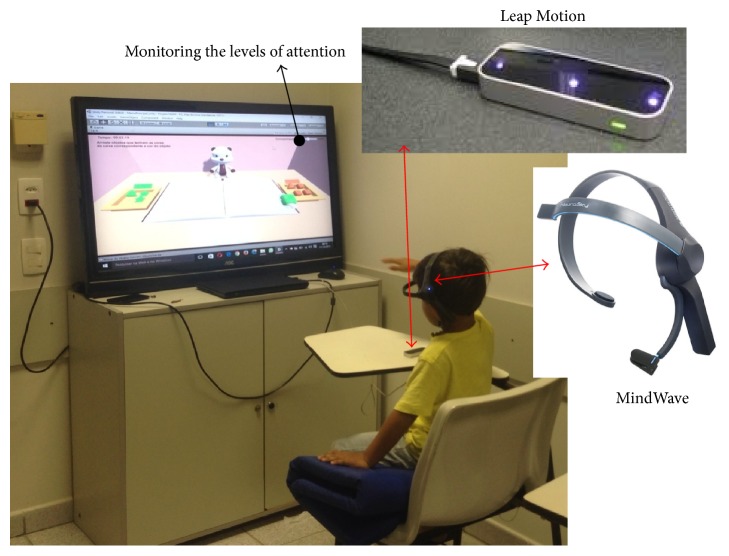
Interaction of the proposed virtual environments with a child.

Figure 5 shows the use of the alternative treatment tool in a 5-year-old child, considering their behavior during their interaction with the virtual environment through the MindWave and Leap Motion sensors, as well as the usability of the tool for possible adjustments according to the difficulties presented by the child. Initially, we observed that the children tend to face difficulties in task execution, due the proximity of the manipulated object relative to the virtual hand; that is, the virtual hand did not have enough space to move. To solve this problem, the depth scale was adjusted from the virtual environment scenario retreating all scenes in the “*Z*” axis, and, from this adjustment, the virtual hand had more space and could be manipulated to perform the tasks.

### 3.1. Validation of the Proposed Virtual Environment

For validation of the virtual environment, a questionnaire based on the perceptions of specialists at the Occupational Therapy Center of NAMI was considered. According to Pfleeger [33], Wohlin et al. [34], and Wohlin et al. [35], a questionnaire should be used before a technique or tool is submitted to quantitative analysis. The aim of the questionnaire was to assure that important issues related to the study were considered as well as to characterize expectations, perceptions, and opportunities about the real use of the proposed virtual environment as a complementary tool to traditional treatment of children with cerebral palsy.

The questionnaire was carried out after the conclusion of the virtual environment, during an experimental presentation to specialists. The questionnaire was based on activities already undertaken in the conventional treatment, in order to make adjustments and estimate risks. At the end of this process, the system was presented to children.

The questionnaire was composed of 14 questions (11 objective and 3 subjective questions), and was filled out by 8 specialists after exposure to the proposed virtual environment. We found that 87.5% of the specialists considered it highly probable that the proposed virtual environment can assist in the motor rehabilitation of the upper limbs. 50% of experts considered it highly probable that the alternative tool will aid in the cognitive evolution of the patient and enhance the motivation of the patient during the treatment. 37.5% and 62.5% of the experts considered it highly likely and very likely, respectively, that the proposed virtual environment can increase the levels of concentration/attention of children during the treatment, with a positive impact on the evolution of motor and cognitive functions. 12.5%, 25.0%, and 62.5% of the specialists found it extremely likely, very likely, or unlikely, respectively, that the proposed virtual environment can avoid/reduce treatment withdrawal. 37.5% considered it extremely likely, 37.5% considered it very likely, and 25% considered it unlikely that the proposed virtual environment can positively influence the neuroplasticity of children. 87.5% of the experts pointed out that it is very likely and 12.5% pointed out that it is very unlikely that the activities presented in each Phase of the environment/game proposed are aligned with the aims of traditional treatment. When asked whether the virtual environment can jeopardize the motor and cognitive evolution of the children, 12.5% and 87.5% of professionals highlighted that this is very unlikely or not likely, respectively. 25% and 75% of the respondents considered it very likely or unlikely, respectively, that the children may reject using the brainwave sensor. Regarding the possible rejection of using Leap Motion, 12.5% considered it very likely and 87.5% considered it very unlikely to occur.

With regard to the subjective questions, the experts pointed out the following.

*Positive Points*. Positive points are as follows: playful game, with different levels of difficulty, friendly environment, ease of developing new phases with different levels, access to technology, new possibilities of motor and cognitive stimulation, new environment for treatment, motivation, and interest of the children, enhancement of the attention level, differentiated alternative, and interesting and current tool.

*Negative Points*. Negative points are as follows: concern about overuse, lack of interest in the conventional treatment, possible rejection of the “MindWave” sensor, despite the fact that its use is optional, and difficulty in handling the “Leap Motion” sensor due to the motor restrictions of children, besides other factors.

*Improvements Possibilities*. Improvement possibilities are as follows: adapted chair or another support for children to manipulate the virtual environment using Leap Motion without unnecessary effort, considering the whole body, for example, using sensor Kinect, and updating the environment often to avoid children's disengagement from the task.

**Figure 6:**
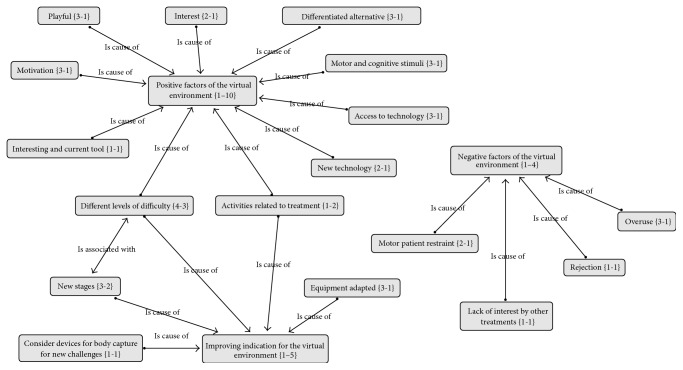
Analysis of the subjective questionnaire answers.

The data analysis software ATLAS.ti [36] was used to assess the subjective questionnaire answers by checking how often specific items were listed among the responses.  Figure 6 shows the causes that are related to the subjective answers: positive points of the virtual environment, negative points of the virtual environment, and improvement of the virtual environment. It is worth noting the number of times that the positive factor is interconnected with other items, showing great affinity with other answers, differently from negative factors, which have very little connection with the answers.

In consultation with experts in Occupational Therapy and Physical Therapy we established that the group of potential users should be selected based on the activities carried out by the expert responsible for monitoring the specific group, who could assess whether the patients could use the proposed virtual environment, given their motor and cognitive levels. The criteria for patients to be included in the study were the following: ataxic, spastic, and dyskinetic cerebral palsy; motor development sufficient for the accomplishment of movements such as walking, running, and jumping; absent or mild spasticity; speed-dependent muscle tone with exacerbation of deep tendon reflexes resulting from hyperexcitability of the stretch reflex; and absence of hypersensitivity to light and attendance to treatment [37].

## 4. Conclusions and Future Perspectives

In this work, we present the development of a new game in virtual reality as an alternative tool to aid the treatment of motor and cognitive impairments in children with cerebral palsy. The integration of the virtual environment with “Leap Motion” was shown to be quite feasible when applied to the rehabilitation of patients with CP, and its integration with MindWave offers a real-time analysis, in which the specialist, to follow the child during the use of this technology, can correlate the level of attention with the evolution of the clinical condition. We found that it was possible to analyze, through testing one child, the possible difficulties and/or facilities of using the proposed method. The application of a questionnaire to specialists allowed assessing the effectiveness and efficiency of the virtual environment, enabling its use in rehabilitation. The specialists were highly optimistic about including the use of the virtual environment in addition to the traditional treatments, as an alternative playful tool for cognitive and motor rehabilitation among children.

As future work, the proposed environment will be included as an auxiliary tool in NAMI, among the complementary activities during the treatment of children with cerebral palsy, analyzing real effectiveness on patient outcomes during a six-month period, periodically assessing the clinical condition of each patient involved.

We intend to analyze the performance of the proposed system for the treatment of other diseases demanding cognitive and motor rehabilitation. If necessary, we will increase the amount of activities/Phases so as to allow additional levels of difficulty, offering patients new challenges within their limitations. Finally, we plan to assign intelligence to the proposed system, making it able to provide novel changes according to the patient's clinical evolution.

## Figures and Tables

**Box 1:**
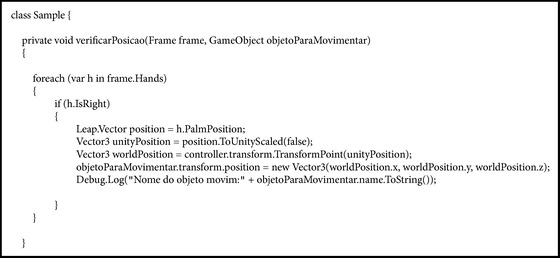


**Box 2:**
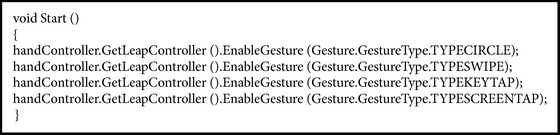


**Box 3:**
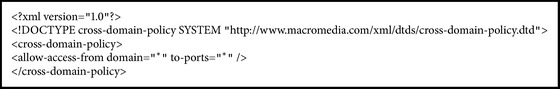


**Box 4:**